# miRNAs cooperate in apoptosis regulation during *C. elegans* development

**DOI:** 10.1101/gad.288555.116

**Published:** 2017-01-15

**Authors:** Ryan Sherrard, Sebastian Luehr, Heinke Holzkamp, Katherine McJunkin, Nadin Memar, Barbara Conradt

**Affiliations:** 1 Center for Integrated Protein Science Munich – CIPSM, Department Biology II, Ludwig-Maximilians-University Munich, Planegg-Martinsried 82152, Germany;; 2 Program in Molecular Medicine, RNA Therapeutics Institute, University of Massachusetts Medical School, Worcester, Massachusetts 01606, USA

**Keywords:** miRNA, programmed cell death, BH3-only, development, embryo, *C. elegans*

## Abstract

Programmed cell death occurs in a highly reproducible manner during *Caenorhabditis elegans* development. We demonstrate that, during embryogenesis, miR-35 and miR-58 *bantam* family microRNAs (miRNAs) cooperate to prevent the precocious death of mothers of cells programmed to die by repressing the gene *egl-1*, which encodes a proapoptotic BH3-only protein. In addition, we present evidence that repression of *egl-1* is dependent on binding sites for miR-35 and miR-58 family miRNAs within the *egl-1* 3′ untranslated region (UTR), which affect both mRNA copy number and translation. Furthermore, using single-molecule RNA fluorescent in situ hybridization (smRNA FISH), we show that *egl-1* is transcribed in the mother of a cell programmed to die and that miR-35 and miR-58 family miRNAs prevent this mother from dying by keeping the copy number of *egl-1* mRNA below a critical threshold. Finally, miR-35 and miR-58 family miRNAs can also dampen the transcriptional boost of *egl-1* that occurs specifically in a daughter cell that is programmed to die. We propose that miRNAs compensate for lineage-specific differences in *egl-1* transcriptional activation, thus ensuring that EGL-1 activity reaches the threshold necessary to trigger death only in daughter cells that are programmed to die.

## Abstract (precis)

Sherrard et al. demonstrate that, during embryogenesis, miR-35 and miR-58 *bantam* family miRNAs cooperate to prevent the precocious death of mothers of cells programmed to die by repressing the gene *egl-1*, which encodes a proapoptotic BH3-only protein.

Programmed cell death (apoptosis) is crucial for proper animal development, and perturbations in the cell death pathway or its regulation have been implicated in disorders such as autoimmune diseases and cancer (for review, see Conradt 2009; Fuchs and Steller 2011). In the nematode *Caenorhabditis elegans*, 131 cells are eliminated by programmed cell death in the developing hermaphrodite, and 113 of these deaths occur during embryogenesis (Sulston and Horvitz 1977; Sulston et al. 1983). Moreover, 98 of the 113 embryonic cell deaths affect cells derived from the AB blastomere (i.e., occur in the “AB lineage”). Cells programmed to die during *C. elegans* development are generally the result of an asymmetric cell division and exhibit distinct characteristics, such as detachment from surrounding cells and formation of a round, optically refractile cell corpse. Ultimately, cell corpses are engulfed and degraded by a neighboring cell (Hedgecock et al. 1983; Sulston et al. 1983). The core genetic pathway that triggers apoptotic cell death is conserved from nematodes to mammals and, in *C. elegans*, consists of four genes, *egl-1*, *ced-9, ced-4*, and *ced-3*, which act in a simple linear pathway (for review, see Horvitz 2003; Lettre and Hengartner 2006; Conradt et al. 2016). The *egl-1* gene encodes a proapoptotic BH3-only protein, which binds to the anti-apoptotic Bcl-2-like protein CED-9 in cells programmed to die. This binding causes the release of the Apaf-1-like adaptor protein CED-4 from CED-9. CED-4 then forms the apoptosome, which mediates activation of the caspase CED-3. CED-3 then induces the processes necessary for the swift demise of the cell. Loss-of-function mutations in either of the proapoptotic genes (*egl-1*, *ced-4*, or *ced-3*) or a gain-of-function mutation in the anti-apoptotic gene *ced-9* block nearly all programmed cell deaths in *C. elegans* (Ellis and Horvitz 1986; Hengartner et al. 1992; Conradt and Horvitz 1998).

As the most-upstream component of the central apoptotic pathway, the BH3-only protein EGL-1 must be tightly controlled to avoid inappropriate cell death (for review, see Nehme and Conradt 2008). One level at which EGL-1 activity is controlled is the transcriptional level. Mutations in *cis*-acting elements of the *egl-1* locus result in the misexpression of *egl-1* and, consequently, changes in the highly reproducible pattern of programmed cell death during development (Conradt and Horvitz 1999; Hirose et al. 2010). In addition, transcriptional reporters have demonstrated that the *egl-1* gene is most highly expressed in cells programmed to die (Conradt and Horvitz 1999; Thellmann et al. 2003; Liu et al. 2006; Potts et al. 2009; Hirose et al. 2010; Winn et al. 2011; Hirose and Horvitz 2013; Jiang and Wu 2014; Wang et al. 2015). Furthermore, genetic studies revealed that *egl-1* transcription is governed by lineage-specific transcription factors, which control the death of individual, often bilaterally symmetric, cells (for review, see Conradt et al. 2016). For example, a heterodimer of the basic helix–loop–helix (bHLH) transcription factors HLH-2 Daughterless and HLH-3 Achaete-scute (HLH-2/HLH-3) binds to a specific *cis*-acting element of the *egl-1* locus [referred to as B(*egl-1*)] and is required for *egl-1* transcriptional activation in the left and right NSM sister cells, which are programmed to die (Thellmann et al. 2003). In the left and right NSMs, which are programmed to survive, the Snail-like zinc finger transcription factor CES-1 antagonizes HLH-2/HLH-3 function, thereby preventing *egl-1* transcriptional activation (Metzstein and Horvitz 1999; Thellmann et al. 2003; Hatzold and Conradt 2008).

Little is known about the post-transcriptional or post-translational regulation of EGL-1 activity. In mammals, at least eight BH3-only proteins exist, and their activities are regulated at various levels (for review, see Happo et al. 2012), including transcriptional (Oda et al. 2000; Nakano and Vousden 2001) and post-translational (Zha et al. 1996; Li et al. 1998) mechanisms. Mammalian BH3-only genes are also subject to post-transcriptional regulation via microRNAs (miRNAs). It has been demonstrated, for example, that the 3′ untranslated region (UTR) of *Bim* mRNA is the target of several miRNAs, which act to down-regulate and fine-tune *Bim* expression (Ventura et al. 2008; Kole et al. 2011; Qian et al. 2011; Pernaute et al. 2014; for review, see Sionov et al. 2015).

In this study, we investigate the roles in programmed cell death during *C. elegans* development of the miR-35 and miR-58 families of miRNAs (Lau et al. 2001; Kato et al. 2009; Alvarez-Saavedra and Horvitz 2010; Wu et al. 2010). The miR-35 family consists of eight members, collectively referred to as miR-35-42, which are found at high levels in oocytes and throughout early and mid-stage embryos but whose levels decline post-gastrulation (>350 embryonic nuclei) (Stoeckius et al. 2009; Alvarez-Saavedra and Horvitz 2010; Isik et al. 2010; Wu et al. 2010). The loss of all eight members in *mir-35-41(nDf50) mir-42(nDf49)* mutants (referred to as “*mir-35* family mutants”) results in 100% embryonic lethality (Alvarez-Saavedra and Horvitz 2010). Animals lacking only the seven miRNAs miR-35-41 are viable but display altered RNAi sensitivity, reduced fecundity, and impaired responsiveness to hypoxia (Massirer et al. 2012; McJunkin and Ambros 2014; Kagias and Pocock 2015). The miR-58 family is homologous to *bantam* miRNA in *Drosophila* and consists of six members: four members that are found at increasing levels throughout embryos starting from mid-stage embryogenesis (miR-80, miR-58.1, miR-81, and miR-82) and two members that are found only at low levels at any stage of development (miR-58.2 and miR-2209.1) (Stoeckius et al. 2009; Alvarez-Saavedra and Horvitz 2010; Isik et al. 2010; Wu et al. 2010). The loss of all four abundant members in *mir-80(nDf53); mir-58.1(n4640); mir-81-82(nDf54)* mutants (referred to as “*mir-58* family mutants”) results in reduced body and brood size as well as defects in locomotion and dauer formation (Alvarez-Saavedra and Horvitz 2010). A previous study demonstrated that *egl-1* mRNA is an in vitro target of both miR-35 and miR-58 family miRNAs (Wu et al. 2010). Furthermore, it was proposed that miR-35 and miR-58 family miRNAs cause translational repression of *egl-1* mRNA by mediating its deadenylation (Wu et al. 2010). Another study reported that the loss of the *mir-58* family causes impairment of germline apoptosis; however, a target was not identified (Subasic et al. 2015). Therefore, to date, direct regulation of the central apoptotic pathway by miRNAs in vivo has not been demonstrated.

Here, we show that loss of the *mir-35* family of miRNAs leads to the presence of abnormally large cell corpses in embryos and that this phenotype is enhanced in *mir-35 mir-58* double-family mutants. These large corpses correspond to mothers and sisters of cells programmed to die and hence cells that normally survive. Furthermore, we demonstrate that the “precocious” and “collateral” death of mothers and sisters, respectively, is dependent on *egl-1* BH3-only and that *egl-1* mRNA is present in these mothers and sisters. Finally, we examined the temporal dynamics of *egl-1* mRNA in cell death lineages and propose that *egl-1* mRNA is targeted by miR-35 and miR-58 family miRNAs in order to avoid traversal of a cellular threshold that would trigger death.

## Results

### *mir-35* family mutants exhibit a large cell corpse phenotype

**Figure 1.**
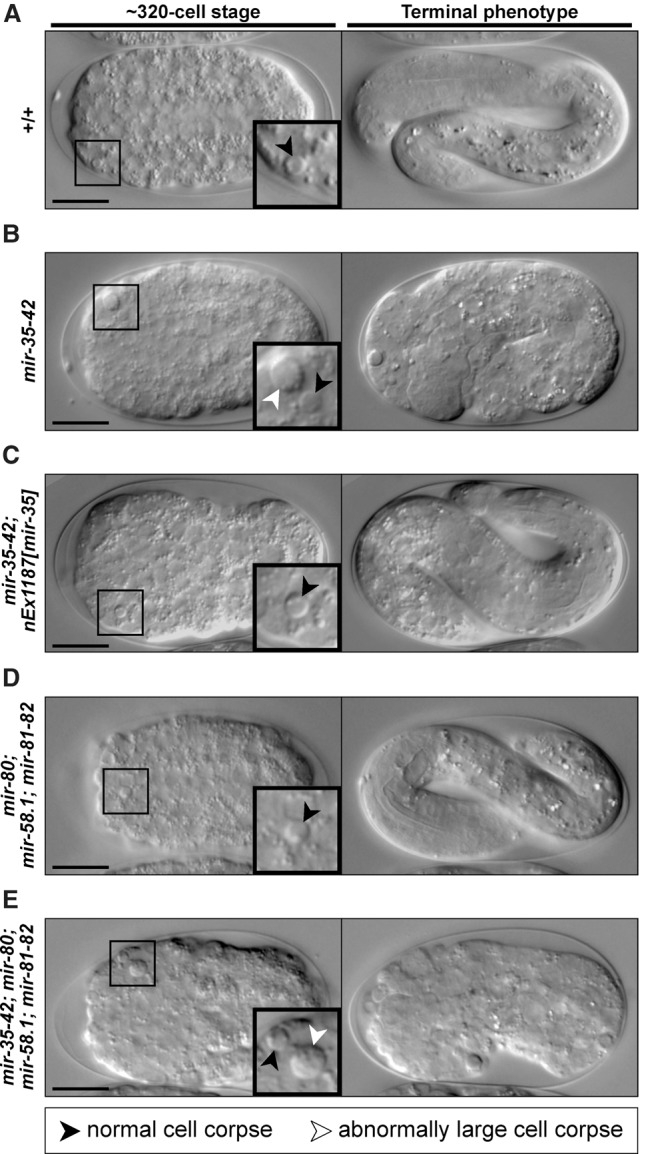
Embryos lacking miR-35 family miRNAs exhibit a large cell corpse phenotype. (*A–E*) Differential interface contrast (DIC) images of embryos of the genotypes +/+ (*A*), *mir-35-41(nDf50) mir-42(nDf49)* (*B*), *mir-35-41(nDf50) mir-42(nDf49); nEx1187* [*mir-35 (genomic)+sur-5*::*gfp*] (*C*), *mir-80(nDf53); mir-58.1(n4640); mir-81-82(nDf54)* (*D*), and *mir-35-41(nDf50) mir-42(nDf49); mir-80(nDf53); mir-58.1(n4640); mir-81-82(nDf54)* (*E*). For each panel, a developing embryo (∼320-cell stage) is depicted at the *left*, with *insets* showing representative cell corpses. Normal cell corpses (black arrowheads) are present in all genetic backgrounds, but abnormally large cell corpses (white arrowheads) are also present (see *B*,*E*). The terminal phenotype of each embryo is shown at the *right*; those in *A*, *C*, and *D* survived to hatching, whereas those in *B* and *E* arrested. Bars, 10 µm.

**Figure 2.**
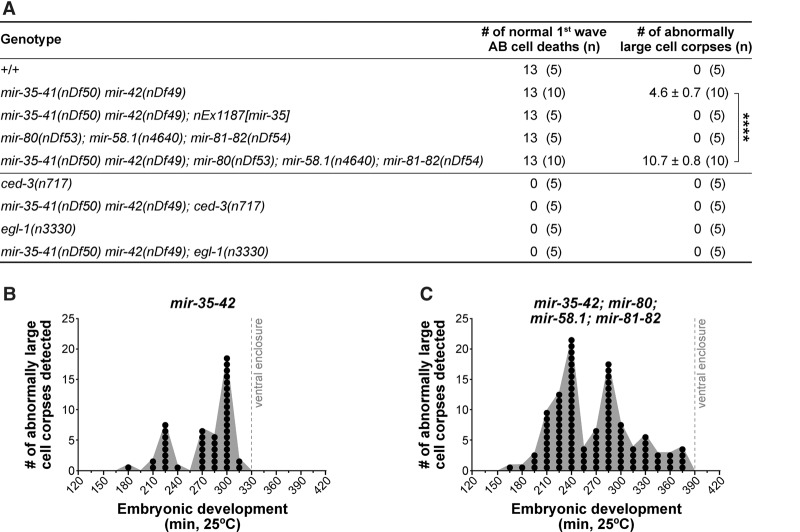
Inappropriate cell death in *mir-35* family mutants is enhanced upon loss of the *mir-58* family and is dependent on the central apoptotic pathway. (*A*) The number of first-wave AB cell deaths was scored per embryo of each genotype. *n* = 5 or 10, as indicated. The number of abnormally large corpses per embryo was also scored until ventral enclosure. Values are averages ± SEM when applicable. (****) *P* ≤ 0.0001 via Student's *t*-test. (*B*,*C*) The number of large cell corpses forming over time in *mir-35-41(nDf50) mir-42(nDf49)* mutants (*B*) and *mir-35-41(nDf50) mir-42(nDf49); mir-80(nDf53); mir-58.1(n4640); mir-81-82(nDf54)* mutants (*C*) until ventral enclosure. Data are based on the time of initial detection for each of the 46 and 107 large cells corpses in *B* and *C*, respectively. Each circle represents the detection of a single large cell corpse. Detection times were grouped into 15-min bins (i.e., intervals). *n* = 10 embryos for each genotype.

To identify cell death abnormalities in miRNA family mutants, we analyzed embryonic development using four-dimensional (4D) microscopy (Schnabel et al. 1997, 2006). We first analyzed wild-type (+/+) embryos and detected the 13 cell deaths of the “first wave of AB cell death” (Figs. 1A, 2A; Supplemental Fig. S1). As expected, dying cells rounded up to form refractile corpses ∼2.5 µm in diameter that were engulfed by neighboring cells. Next, we analyzed *mir-35* family mutants [*mir-35-41(nDf50) mir-42(nDf49)*] prior to embryonic arrest. We identified the 13 cell deaths of the first wave of AB cell death (Figs. 1B, 2A); however, in addition to the normal cell corpses, we also detected corpses with diameters ∼1.5-fold larger than normal (Fig. 1B). These large corpses displayed the typical attributes of cell corpses; however, not all were eventually engulfed by neighboring cells. Instead, they often persisted or were extruded from the developing embryo (Supplemental Fig. S2). We quantified the number of large cell corpses per embryo until a predetermined developmental endpoint (ventral enclosure of the epidermal cells; ∼330 min at 25°C) and detected an average of 4.6 large corpses per embryo (Fig. 2A). Large cell corpses were never detected earlier than ∼180 min of embryonic development, and their formation became more frequent as embryogenesis progressed, peaking at ∼225 min and again at ∼300 min (Fig. 2B).

This large cell corpse phenotype has not been characterized previously in *mir-35* family mutants. Therefore, we tested whether the phenotype could be rescued by a transgene (*nEx1187*) that carries *mir-35* (Alvarez-Saavedra and Horvitz 2010). Since transgenic embryos contained only normal-sized cell corpses (Figs. 1C, 2A), we attribute the large cell corpse phenotype specifically to the loss of miR-35 family miRNAs.

### The large cell corpse phenotype of *mir-35* family mutants is enhanced by the loss of the *mir-58* family

We next assessed cell death in *mir-58* family mutants [*mir-80(nDf53); mir-58.1(n4640); mir-81-82(nDf54)*]. Normal cell corpses were detected throughout embryogenesis, but no large cell corpses were observed, and all embryos survived to hatching (Figs. 1D, 2A). Finally, we generated a strain lacking both miR-35 and miR-58 family miRNAs [*mir-35-41(nDf50) mir-42(nDf49); mir-80(nDf53); mir-58.1(n4640); mir-81-82(nDf54)*] and examined embryos for the large cell corpse phenotype. As observed in *mir-35* family mutants, both normal and large cell corpses were present in *mir-35 mir-58* double-family mutants, and all embryos arrested (Fig. 1E). We quantified the number of large cell corpses per embryo prior to ventral enclosure (∼390 min at 25°C in *mir-35 mir-58* double-family mutants) and detected an average of 10.7 large corpses per embryo (Fig. 2A), a 2.3-fold increase over *mir-35* family mutants. Large corpses were never detected earlier than ∼160 min of embryonic development, and, as in *mir-35* family mutants, the formation of large corpses increased over time, peaking at ∼240 min and again at ∼285 min (Fig. 2C). Therefore, the large cell corpse phenotype in the *mir-35* family mutant is enhanced by the loss of the *mir-58* family.

### miR-35 family miRNAs act upstream of or in parallel to *egl-1* BH3-only

The large cell corpses observed in *mir-35* family mutants and *mir-35 mir-58* double-family mutants displayed the typical attributes of cells programmed to die in *C. elegans*. Therefore, we tested whether the formation of these large cell corpses requires the central apoptotic pathway. The loss of either *ced-3* caspase or *egl-1* BH3-only blocks most programmed cell deaths in *C. elegans* (Ellis and Horvitz 1986; Conradt and Horvitz 1998), including the 13 cell deaths of the first wave of AB cell death (Fig. 2A). Our analysis of *mir-35-42; ced-3* and *mir-35-42; egl-1* double mutants revealed that both normal-sized and large cell corpses were absent during embryogenesis. This indicates that miR-35 family miRNAs act upstream of or in parallel to *egl-1* to suppress the appearance of abnormally large cell corpses. Of note, the loss of either *ced-3* or *egl-1* did not rescue embryonic lethality in *mir-35* family mutants (Supplemental Fig. S3), indicating that the large cell corpse phenotype is not the underlying cause of embryonic arrest.

### The 3′ UTR of *egl-1* causes repression of reporter gene expression in a *mir-35* and *mir-58* family-dependent manner

**Figure 3.**
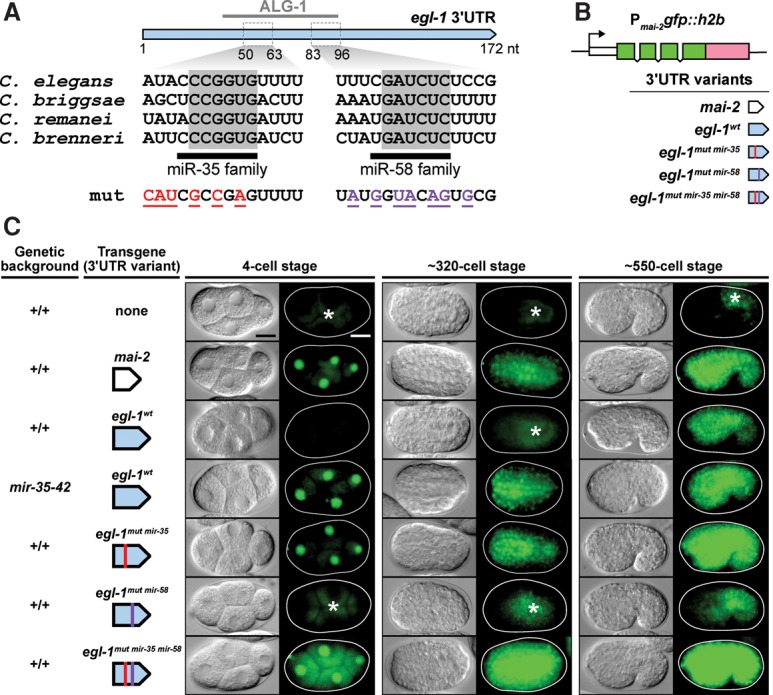
The 3′ UTR of *egl-1* is a target of miR-35 and miR-58 family miRNAs in vivo. (*A*) The 3′ UTR of *egl-1* is illustrated in blue, with a reported *C. elegans* Argonaut ALG-1-binding site from a recent study (Grosswendt et al. 2014) indicated *above*. Sequences are given for predicted miR-35 family-binding sites and miR-58 family*-*binding sites, and conserved bases within each site are highlighted in gray across four *Caenorhabditis* species. The mutated sequences corresponding to *egl-1*^*mut mir-35*^ (red) and *egl-1*^*mut mir-58*^ (purple) are shown (for complete mutated sequences, see the Materials and Methods). (*B*) A schematic representation of the 3′ UTR reporters constructed for this study. (*C*) Analysis of single-copy 3′ UTR reporter expression during embryogenesis. The genetic background and transgene under investigation are indicated at the *left* of each image sequence, which shows representative embryos from three developmental stages. For each embryo, a DIC image (*left*) and GFP image (*right*) are shown. The following alleles were used: *bcSi25* [P*_mai-2_gfp::h2b::mai-2 3′ UTR*], *bcSi26* [P*_mai-2_gfp::h2b::egl-1^wt^ 3*′ UTR], *bcSi27* [P*_mai-2_gfp::h2b::egl-1^mut mir-35^ 3′ UTR*], *bcSi46* [P*_mai-2_gfp::h2b::egl-1^mut mir-35^ 3′ UTR*], and *bcSi47* [P*_mai-2_gfp::h2b::egl-1^mut mir-35 mir-58^ 3′ UTR*]. Asterisks indicate autofluorescence. Transgenic strains were homozygous for *unc-119(ed3)* and the *cb-unc-119(+)* selection marker. Bars, 10 µm.

The 3′ UTR of *egl-1* is predicted to harbor multiple miRNA-bindings sites, including one for miR-35 family miRNAs and one for miR-58 family miRNAs (Fig. 3A; TargetScanWorm release 6.2; Jan et al. 2011). In addition, both sites are conserved across at least three other nematode species and were found to associate with *C. elegans* Argonaut ALG-1 in iPAR-CLIP (in vivo photoactivatable ribonucleoside-enhanced cross-linking and immunoprecipitation) experiments, suggesting that they are likely targets of the RNA-induced silencing complex (RISC) (Grosswendt et al. 2014). To determine whether the *egl-1* 3′ UTR can suppress the expression of a reporter gene in vivo, we generated five different reporter constructs (Fig. 3B). Each of the reporters contained a *gfp::histone h2b* fusion gene (*gfp::h2b*) driven by the *mai-2* promoter (P*_mai-2_gfp::h2b*) (see the Materials and Methods; Ichikawa et al. 2006). The five reporters differed only in their 3′ UTRs. The first reporter contained the 3′ UTR of the *mai-2* gene, which lacks predicted binding sites for miR-35 and miR-58 family miRNAs (*mai-2* 3′ UTR). The remaining reporters contained the wild-type *egl-1* 3′ UTR (*egl-1*^*wt*^ 3′ UTR) or mutant *egl-1* 3′ UTRs, in which the predicted miR-35 family miRNA-binding site (*egl-1*^*mut mir-35*^ 3′ UTR), the predicted miR-58 family miRNA-binding site (*egl-1*^*mut mir-58*^ 3′ UTR), or both (*egl-1*^*mut mir-35 mir-58*^ 3′ UTR) were mutated (Fig. 3A, B). The expression pattern of each transgenic construct was then analyzed at the single-copy level (MosSCI [Mos1 transposase-mediated single-copy gene insertion] alleles) in four-cell, ∼320-cell (mid), and ∼550-cell (late) stage embryos. The *mai-2* 3′ UTR transgene displayed ubiquitous expression at all embryonic stages examined (Fig. 3C). In contrast, the expression of the *egl-1*^*wt*^ 3′ UTR transgene was markedly suppressed in all cells in four-cell and ∼320-cell stage embryos; however, expression was detected in ∼550-cell stage embryos (Fig. 3C). (This derepression of transgene expression in older embryos most likely reflects the decrease in *mir-35-42* expression observed after gastrulation [Stoeckius et al. 2009; Alvarez-Saavedra and Horvitz 2010; Isik et al. 2010; Wu et al. 2010].) The *egl-1*^*mut mir-35*^ 3′ UTR transgene as well as the *egl-1*^*wt*^ 3′ UTR transgene in a *mir-35-42* mutant background exhibited ubiquitous expression that was indistinguishable from that of the *mai-2* 3′ UTR transgene. Finally, mutating the binding site for miR-58 family miRNAs had no detectable effect on the repression of an otherwise wild-type transgene (*egl-1*^*mut mir-58*^) but slightly increased expression of the *egl-1*^*mut mir-35*^ transgene (*egl-1*^*mut mir-35 mir-58*^). Therefore, we conclude that miRNAs of the miR-35 family target the *egl-1* 3′ UTR in a site-specific manner to repress expression of the *egl-1* gene and that miRNAs of the miR-58 family may act to enhance this repression.

miRNAs repress target gene expression by causing translational inhibition and/or mRNA decay (for review, see Huntzinger and Izaurralde 2011). To determine which of these two mechanisms is responsible for repression of *gfp::h2b* expression in the case of the *egl-1*^*wt*^ 3′ UTR transgene, we used single-molecule RNA fluorescent in situ hybridization (smRNA FISH) to directly probe *gfp::h2b* mRNA in transgenic embryos. Compared with embryos carrying the *mai-2* 3′ UTR transgene, we found that embryos carrying the *egl-1*^*wt*^ 3′ UTR transgene had significantly reduced copy numbers of *gfp::h2b* mRNA (Supplemental Fig. S4A,B). Based on this observation, we propose that miR-35 and miR-58 family miRNAs repress *egl-1* expression by causing both translational inhibition and decay of *egl-1* mRNA.

### Mothers and sisters of cells normally programmed to die undergo apoptosis in *mir-35* family mutants

**Figure 4.**
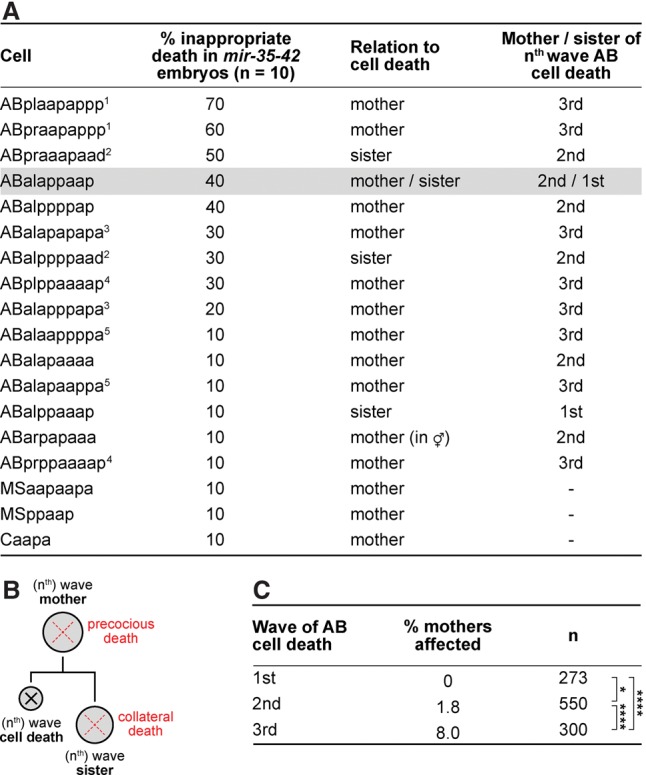
Abnormal cell death in *mir-35* family mutants affects mothers and sisters of programmed cell death. (*A*) The identities of 46 larges corpses present across 10 *mir-35-41(nDf50) mir-42(nDf49)* embryos were determined as described in the Materials and Methods. Numerical superscripts 1–5 indicate pairs of bilaterally symmetric cells. (*B*) Lineage representation of “precocious death” affecting mother cells and “collateral death” affecting sister cells. (*C*) The average percentage of precociously dying mothers was determined per embryo for the three waves of AB cell death according to data presented in *A*. (*) *P* < 0.05; (****) *P* < 0.0001 via Fisher’s exact test.

Abnormally large cell corpses have been observed previously in some *C. elegans* mutants (Hengartner et al. 1992; Sugimoto et al. 2001; Frank et al. 2005), and their appearance has been attributed to the death of physically larger cells, which might result from a prematurely active cell death program or a reversal of polarity in the mother of a cell programmed to die. Therefore, using 4D microscopy and lineage analyses, we determined the identities of all 46 large cell corpses that we had detected in 10 *mir-35* family mutant embryos (Fig. 2A). In each case, we found that the large corpse was formed by a cell that was not normally programmed to die; however, each was closely related to a programmed cell death (Fig. 4A). Specifically, all large cell corpses could be classified as either the mother of a cell death (37 out of 46; ∼80%) or the sister of a cell death (13 out of 46; ∼28%). (One inappropriately dying cell, ABalappaap, which died in four of 10 embryos [40% inappropriate death in *mir-35-42* embryos], is both a sister and a mother of a cell death and is therefore considered in both classifications.) Hence, we consider these inappropriate deaths to be “precocious” in the case of mother cells and “collateral” in the case of sister cells (Fig. 4B). The precocious death of mother cells always occurred prior to the expected time of division, and divisions giving rise to collaterally dying sister cells exhibited normal timing and polarity (data not shown). Thus, our data support a prematurely or collaterally active central apoptotic pathway as the cause of inappropriate cell death in *mir-35* family mutants, affecting mothers and sisters of programmed cell deaths.

### Lineage and developmental stage affect the penetrance of inappropriate cell deaths in *mir-35* family mutants

ABalappaap dies in 40% of *mir-35* family mutants (Fig. 4A). We specifically examined ABalappaap in *mir-35 mir-58* double-family mutants and discovered that this cell dies precociously in 80% of embryos (*n* = 10), which is double the penetrance observed in *mir-35* family mutants (Fig. 4A). This provides further evidence that inappropriate death, resulting in the formation of large cell corpses, is enhanced in *mir-35 mir-58* double-family mutants.

The 98 programmed cell deaths of the AB lineage can be classified into three temporal “waves” (Supplemental Fig. S1). The first wave consists of 13 cells that die after the ninth round of embryonic cell division. The second and third waves consist of 55 and 30 cells that die after the 10th and 11th rounds of embryonic cell division, respectively. Notably, mothers of first-wave cell deaths were never found to die precociously (zero of 273) in *mir-35* family mutants (Fig. 4C). Mothers of second-wave cell deaths were modestly affected, with an average of 1.8% dying precociously (10 of 550). However, the greatest impact was on mothers of third-wave cell deaths, with an average of 8% dying precociously (24 of 300). Thus, we conclude that a loss of the *mir-35* family does not trigger the precocious death of mothers of the first wave of cell deaths in the AB lineage, and mothers of the third wave are about four times more likely to die precociously than mothers of the second wave.

### miRNA-dependent control of *egl-1* mRNA copy number is crucial for the survival of ABalappaap

**Figure 5.**
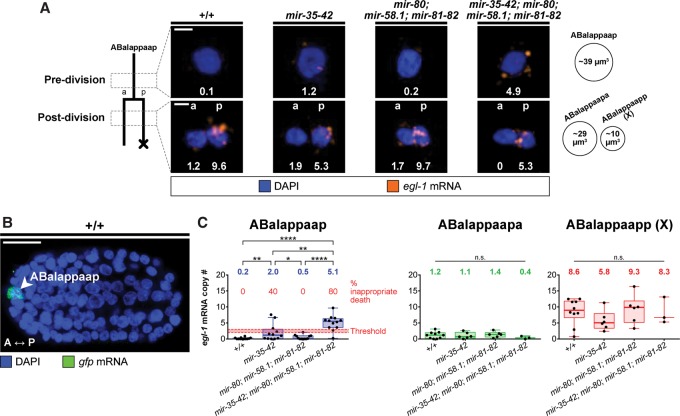
*egl-1* mRNA copy number crosses a threshold to trigger the precocious death of ABalappaap in *mir-35 mir-58* double-family mutants. (*A*) smRNA FISH analysis in the ABalappaap lineage. The lineage is illustrated at the *right*, with the approximate time of each analysis (pre- and post-division) indicated. Representative images of the ABalappaap cell and its daughters are shown for each time point in four genetic backgrounds, as indicated. The *egl-1* mRNA copy number corresponding to each image is given. Nuclei are labeled as belonging to the surviving daughter (anterior [a]) or the dying daughter (posterior [p]). Approximate cellular volumes are given at the *right*. Bars, 2 µm. (*B*) A fixed wild-type embryo showing the ABalappaap cell (arrowhead) as identified by smRNA FISH staining against mRNA transcribed from the P*_unc-3_unc-3::gfp* reporter. The anterior–posterior axis (A ↔ P) is indicated. Bar, 10 µm. (*C*) Quantification of *egl-1* mRNA copy number in the ABalappaap cell and its two daughters (ABalappaapa and ABalappaapp [X]) for each of the indicated genotypes. An estimated threshold for triggering cell death in ABalappaap is represented by the red line. The mean value is given *above* each data set. Box and whiskers were plotted according to Tukey's test. (*) *P* < 0.05; (**) *P* < 0.01; (****) *P* < 0.0001 via Mann-Whitney test. The following alleles were used: *mir-35-41(nDf50)*, *mir-42 (nDf49)*, *mir-80(nDf53)*, *mir-58.1(n4640)*, and *mir-81-82(nDf54).* All strains analyzed carried the extrachromosomal array *xdEx1091* [P_*unc-3*_*unc-3::gfp*+P*_sur-5_rfp*].

Using smRNA FISH, we directly probed *egl-1* mRNA in the ABalappaap lineage in situ. To identify ABalappaap in fixed whole-mount embryos, we used an *unc-3* reporter (P*_unc-3_unc-3::gfp*) (Wang et al. 2015) and concurrently stained *gfp* mRNA (Fig. 5B). Single-cell copy numbers of *egl-1* mRNA were quantified from image stacks obtained by confocal microscopy. Briefly, the total smRNA FISH signal intensity corresponding to ABalappaap was quantified and then divided by the intensity of a single mRNA molecule, thereby yielding a measure of mRNA copy number (see the Materials and Methods; Supplemental Fig. S5). We also measured the volumes of ABalappaap and its daughters (Fig. 5A) and calculated the mean concentrations of *egl-1* mRNA.

We first analyzed ABalappaap in the two genetic backgrounds in which this cell does not die precociously. In both wild-type and *mir-58* family mutant embryos, we found *egl-1* mRNA to be largely absent from ABalappaap, with a mean number of 0.2 and 0.5 copies, respectively (0.01 mRNA copies per cubic micrometer) (Fig. 5A,C; Supplemental Fig. S6A). In the two genetic backgrounds in which ABalappaap dies precociously (*mir-35* family mutants and *mir-35 mir-58* double-family mutants), we measured a significantly larger number of copies of *egl-1* mRNA in ABalappaap, with the highest value found in *mir-35 mir-58* double-family mutants (mean 5.1 copies; 0.13 mRNA copies per cubic micrometer). Since ABalappaap dies in 40% of *mir-35* family mutants (Fig. 2A) and 80% of *mir-35 mir-58* double-family mutants, a corresponding percentage of embryos would be expected to contain copy numbers of *egl-1* mRNA sufficient to trigger death. Therefore, our data suggest that two to three copies of *egl-1* mRNA are sufficient to trigger the precocious death of ABalappaap and hence represent “above threshold” copy numbers of *egl-1* mRNA in this cell (Fig. 5C). In addition, our data demonstrate that miR-35 family miRNAs and miR-58 family miRNAs cooperate in ABalappaap to keep the copy number of *egl-1* mRNA below this threshold.

Next, we analyzed *egl-1* mRNA copy numbers in the daughters of ABalappaap: ABalappaapa, which survives, and ABalappaapp (X), which dies (Fig. 5A,C). (The programmed death of ABalappaapp [X] is dependent on *egl-1* [Wang et al., 2015].) In the surviving daughter, we found similar “below threshold” *egl-1* mRNA copy numbers in all genotypes (Fig. 5C). In contrast, in the dying daughter, we observed similar “above threshold” *egl-1* mRNA copy numbers in all genotypes (Fig. 5C).

### miR-35 and miR-58 family miRNAs affect the level of *egl-1* mRNA in cell death lineages

**Figure 6.**
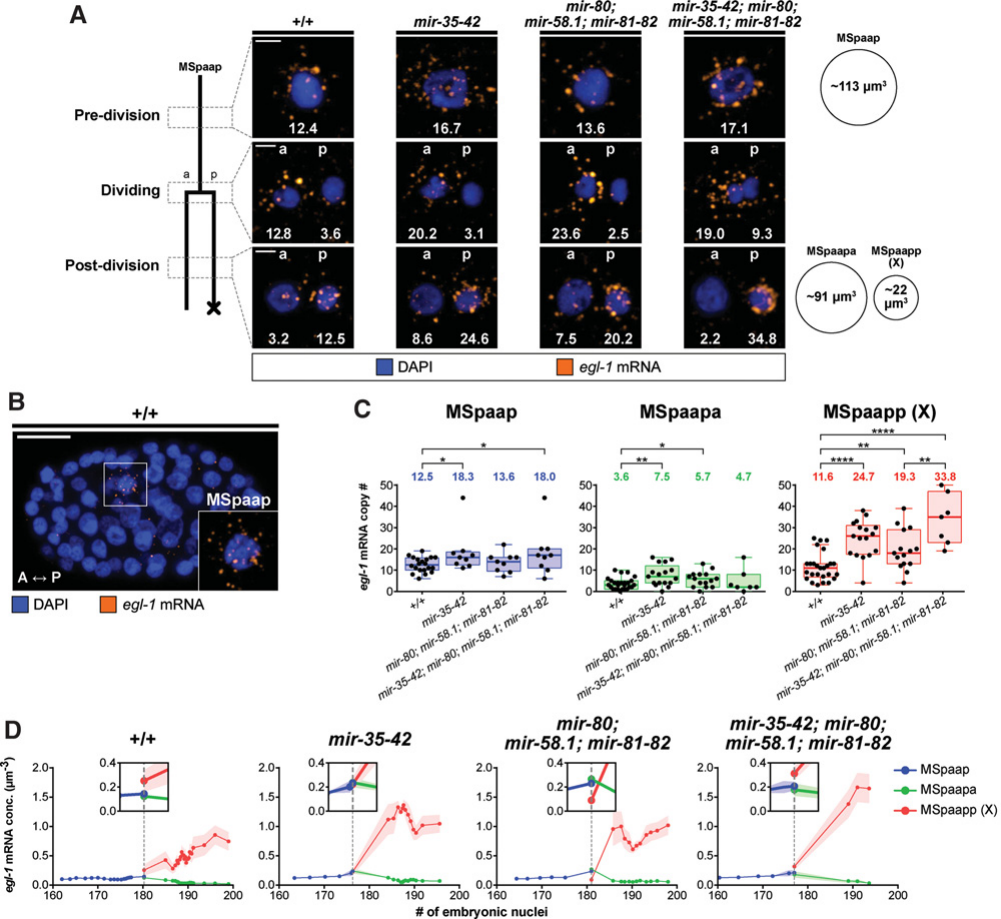
*egl-1* mRNA levels are temporally dynamic in the MSpaap lineage and more abundant in *mir* family mutants. (*A*) smRNA FISH analysis in the MSpaap lineage. The lineage is illustrated at the *right*, with the approximate time of each analysis (predivision, dividing, and post-division) indicated. Representative images of the MSpaap cell and its daughters are shown for each time point in four genetic backgrounds, as indicated. Nuclei are labeled as belonging to the surviving daughter (anterior [a]) or the dying daughter (posterior [p]). The *egl-1* mRNA copy number corresponding to each image is given. Approximate cellular volumes are given at the *right*. Bars, 2 µm. (*B*) A fixed wild-type embryo containing 167 nuclei showing the MSpaap cell (*inset*). The anterior–posterior axis (A ↔ P) is indicated Bar, 10 µm. (C) Quantification of *egl-1* mRNA in the MSpaap cell and its two daughters (MSpaapa and MSpaapp [X]) for each of the indicated genotypes. The mean value is given *above* each data set. Box and whiskers were plotted according to Tukey's test. (*) *P* < 0.05; (**) *P* < 0.01; (****) *P* < 0.0001 via Mann-Whitney test. (*D*) Developmental time course showing *egl-1* mRNA concentration in cells of the MSpaap lineage for the indicated genotypes. Division of the MSpaap cell is indicated by a vertical dashed line. *Insets* show a 2× zoom of the graph at the time of division. Graphs were generated from raw data as a centered moving average of order 5. Shaded areas represent SEM, when applicable. The following alleles were used: *mir-35-41(nDf50)*, *mir-42 (nDf49)*, *mir-80(nDf53)*, *mir-58.1(n4640)*, and *mir-81-82(nDf54)*.

We investigated the copy numbers of *egl-1* mRNA in a second cell death lineage, this time in a lineage in which no effect was observed upon loss of the *mir-35* and/or *mir-58* family (i.e., no inappropriate death). For this purpose, we chose the MSpaap cell and its daughters, since these cells can be identified in fixed whole-mount embryos by DAPI and *egl-1* smRNA FISH signals alone (Fig. 6B). The MSpaap cell divides at the 180-cell stage, giving rise to MSpaapp (X), which is the first cell to undergo programmed cell death during *C. elegans* development. (The programmed death of MSpaapp [X] is dependent on *egl-1* [data not shown].)

We quantified single-cell copy numbers of *egl-1* mRNA at three distinct time points: predivision (MSpaap), dividing (MSpaap), and post-division (MSpaapa and MSpaapp [X]). In addition, we measured the volumes of the cells (Fig. 6A) and calculated the mean concentrations of *egl-1* mRNA. Predivision, *egl-1* mRNA was detected in MSpaap across all genotypes (Fig. 6A). The mean copy number of *egl-1* mRNA in MSpaap in wild type was 12.5 (0.11 mRNA copies per cubic micrometer), and this number increased to as much as 18.0 (0.16 mRNA copies per cubic micrometer) in *mir-35 mir-58* double-family mutants (Fig. 6C; Supplemental Fig. S6B). Since MSpaap was not found to die precociously in any of the mutant backgrounds, we conclude that 0.16 *egl-1* mRNA copies per cubic micrometer is not sufficient to trigger its precocious death. (For comparison, our data suggest that 0.13 *egl-1* mRNA copies per cubic micrometer is sufficient to trigger the precocious death of 80% of ABalappaap cells.) Slight differences in *egl-1* mRNA copy numbers were also observed in the surviving daughter MSpaapa, which also was not found to die collaterally in any of the mutant backgrounds. In contrast, larger and significant differences between the genotypes were observed in the dying daughter MSpaapp (X) (Fig. 6C; Supplemental Fig. S6B). In wild type, the mean concentration of *egl-1* mRNA in this cell was 0.53 mRNA copies per cubic micrometer, which is similar to the mean concentration that we determined for ABalappaapp (X) (0.86 mRNA copies per cubic micrometer) (Supplemental Fig. S6A). *egl-1* mRNA copy numbers and concentration were elevated in the *mir* mutant backgrounds, increasing to as high as 33.8 copies and 1.5 mRNA copies per cubic micrometer in *mir-35 mir-58* double-family mutants. Since our data demonstrate that, in *mir-35* family and/or *mir-58* family mutants, the majority of MSpaapp (X) cells contains copy numbers of *egl-1* mRNA that are significantly larger than in wild type (Fig. 6C), we asked whether this affected their deaths. We measured the time needed for MSpaapp (X) to form a cell corpse in each genetic background and found no difference in the timing of the cell death process (Supplemental Fig. S7).

Next, we plotted *egl-1* mRNA copy numbers (Supplemental Fig. S8) and *egl-1* mRNA concentrations (Fig. 6D) in MSpaap, MSpaapa, and MSpaapp (X) according to the developmental stage of the respective embryo. This revealed the temporal dynamics of *egl-1* mRNA in the three cells. In the mother MSpaap, *egl-1* mRNA levels increased just prior to cell division in all genotypes, presumably reflecting the start of transcriptional up-regulation of the *egl-1* gene (Fig. 6D; Supplemental Fig. S8). In wild type, this amounted to an ∼50% increase, but the increase was larger in *mir-35* and/or *mir-58* family mutants (Supplemental Fig. S8). Furthermore, plotting *egl-1* mRNA concentrations according to developmental stage also revealed that the distribution of *egl-1* mRNA among the descendants of MSpaap reflects the inherent difference in sizes of the two daughter cells and that each “inherits” ∼0.2 *egl-1* mRNA copies per cubic micrometer (Fig. 6D). Therefore, at least in the MSpaap lineage, *egl-1* mRNA does not appear to be segregated asymmetrically into the daughter that is programmed to die.

After the division of MSpaap, the levels of *egl-1* mRNA diminished in the surviving daughter MSpaapa in all genotypes, reaching negligible levels around the 190-cell stage (Fig. 6D; Supplemental Fig. S8). This suggests that the degradation of *egl-1* mRNA in MSpaapa is not dependent on miR-35 and miR-58 family miRNAs. In contrast, in all genotypes, the levels of *egl-1* mRNA increased significantly in the dying daughter Mspaapp (X), presumably as a consequence of *egl-1* transcriptional up-regulation (Fig. 6D; Supplemental Fig. S8). However, the magnitude of this increase varied significantly among the different genetic backgrounds and was most dramatic in *mir-35 mir-58* double-family mutants, peaking at ∼37 copies of *egl-1* mRNA (compared with ∼19 copies of *egl-1* mRNA in the wild type) or ∼1.7 *egl-1* mRNA copies per cubic micrometer (compared with ∼0.9 *egl-1* mRNA copies per cubic micrometer in the wild type) at the 190- to 200-cell stage (Supplemental Fig. S8). Based on these results, we conclude that miR-35 family and miR-58 family miRNAs target *egl-1* mRNA in the MSpaap lineage, although the loss of these miRNA families is not sufficient to trigger inappropriate death.

## Discussion

### miR-35 and miR-58 family miRNAs cooperate to directly repress expression of the BH3-only gene *egl-1* in vivo

**Figure 7.**
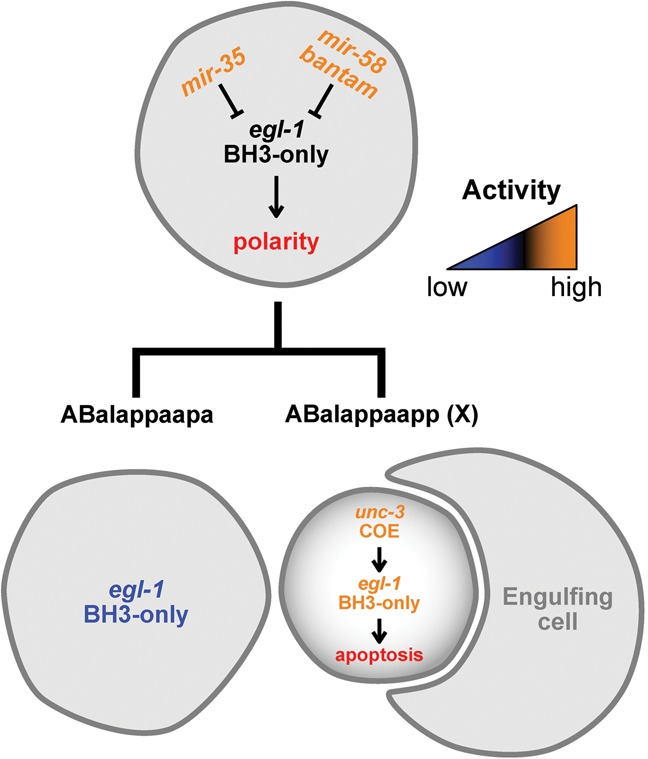
Genetic model of *mir-35-* and *mir-58*-dependent control of *egl-1* BH3-only in the ABalappaap lineage. See the text for details.

We demonstrate that miR-35 and miR-58 family miRNAs cooperate in the ABalappaap and most probably other cell lineages to directly control *egl-1* expression in mothers of cells programmed to die during embryogenesis, thereby preventing their precocious deaths (Fig. 7). “miRNA cooperativity” occurs when the simultaneous targeting of two miRNA-binding sites causes a more than additive effect, and this phenomenon is observed predominantly for closely linked miRNA-binding sites (Grimson et al. 2007; Sætrom et al. 2007). With a distance of 33 nucleotides (nt) separating the miR-35 family-binding site and miR-58 family-binding site, the *egl-1* 3′ UTR is likely the target of cooperative miRNA function, and this is confirmed by our in vivo studies of the ABalappaap lineage. Direct targets have been described recently for the miR-35 family of miRNAs (Liu et al. 2011; Kagias and Pocock 2015) as well as the miR-58 family of miRNAs (de Lucas et al. 2015; Pagano et al. 2015). Furthermore, it has been demonstrated previously that, in *C. elegans*, miRNAs of a particular family can function redundantly (Miska et al. 2007) and/or in combination (Abbott et al. 2005) to control target gene expression. However, at least to our knowledge, the finding that members of the miR-35 and miR-58 family cooperate to directly repress the expression of the BH3-only gene *egl-1* is the first in vivo demonstration of miRNA cooperativity between members of two miRNA families in *C. elegans*.

### miR-35 and miR-58 family miRNAs cause *egl-1* mRNA decay in the ABalappaap and MSpaap cell death lineages

Based on in vitro studies, it has been proposed that, by mediating its deadenylation, miR-35 and miR-58 family miRNAs can cause translational repression of *egl-1* mRNA (Wu et al. 2010). This is confirmed by our in vivo studies of animals carrying the P*_mai-2_gfp::h2b::egl-1 3′ UTR* transgene. (Whether miR-35 and miR-58 family miRNAs also cause translational repression of *egl-1* mRNA generated from the *egl-1* locus is unknown, since we were unable to detect endogenous EGL-1 protein in any genetic background.) Based on our in vivo studies of both the P*_mai-2_gfp::h2b::egl-1 3′ UTR* transgene and *egl-1* mRNA generated from the *egl-1* locus, we propose that miR-35 and miR-58 family miRNAs also mediate *egl-1* mRNA decay. For example, we found that, in the ABalappaap lineage, miR-35 and miR-58 family miRNAs cooperate to control the copy number of *egl-1* mRNA in the mother of a cell programmed to die and thus presumably maintain *egl-1* expression below a threshold that is sufficient to trigger death (Fig. 7). In the MSpaap lineage, in which the loss of the *mir-35* and/or *mir-58* family did not induce precocious or collateral death, miR-35 and miR-58 family miRNAs cooperate to control the copy number of *egl-1* mRNA in the daughter that is programmed to die [MSpaapp (X)] and thereby dampen the transcriptional boost of *egl-1* that occurs in this cell.

Our results raise a number of interesting questions. For example, it is unclear why a mean concentration of 0.13 *egl-1* mRNA copies per cubic micrometer is sufficient to trigger the precocious death of 80% of ABalappaap cells but 0% of MSpaap cells. One possible explanation is that, in the MSpaap lineage, factors other than *egl-1* expression may be limiting. Furthermore, our results indicate that while in the ABalappaap lineage, miR-35 and miR-58 family miRNAs cause *egl-1* mRNA decay predominantly in the mother, in the MSpaap lineage, they act predominantly in the daughter that is programmed to die. As discussed in more detail below, we propose that this reflects the necessity of precisely controling *egl-1* expression and hence EGL-1 activity in a lineage-specific manner. Furthermore, this observation suggests that the activities of miR-35 and miR-58 family miRNAs themselves are precisely controlled in a spatial as well as temporal manner, at least in cell death lineages. How this is accomplished remains to be determined.

### miR-35 and miR-58 family miRNAs adjust lineage-specific differences in *egl-1* transcriptional control

We provide the first in vivo evidence that the activity of the BH3-only gene *egl-1* is regulated at the post-transcriptional level through miRNAs. It is well established that EGL-1 activity is controlled at the transcriptional level (for review, see Nehme and Conradt 2008; Conradt et al. 2016), and it has been proposed that transcription factors that are critical for differentiation in a particular lineage may be used in that same lineage to also cause transcriptional up-regulation of *egl-1* in the daughter that is programmed to die. For example, in the ABalappaap lineage, the COE transcription factor UNC-3 of *C. elegans* is required for both the complete differentiation of the daughter programmed to survive (ABalappaapa) and the death of the daughter that is programmed to die [ABalappaapp (X)] (Wang et al. 2015). [How UNC-3 affects differentiation in ABalappaapa is currently unclear; however, in ABalappaapp (X), UNC-3 triggers cell death by directly activating *egl-1* transcription.] Interestingly, the *unc-3* gene is expressed (and hence UNC-3 protein is presumably present) in not only ABalappaapa and ABalappaapp (X) but also their mother, ABalappaap (Wang et al. 2015). Therefore, we propose that UNC-3 starts to activate *egl-1* transcription in the mother and that miR-35 and miR-58 family miRNAs keep the copy number of *egl-1* mRNAs below the threshold necessary to trigger death in this cell (Fig. 7). The mothers that are affected by the loss of the *mir-35* family are affected to varying degrees (penetrance between 10% and 70%). Furthermore, in different lineages, different transcription factors are responsible for *egl-1* transcriptional regulation. Therefore, we propose that the varying penetrance observed in different lineages of *mir-35* family mutants is a reflection of the lineage-specific regulators of *egl-1* transcription and their functional status in the mothers. Hence, the normal function of miR-35 and miR-58 family miRNAs is to adjust these lineage-specific differences. In support of this notion, the two mothers that die with the highest penetrance, ABplaapappp and ABpraapappp, are mothers of two bilaterally symmetric lineages that most probably use the same transcription factors for *egl-1* transcriptional control.

### miR-35 and miR-58 family miRNAs critically contribute to the precise control of programmed cell death during *C. elegans* development

We demonstrate that miR-35 and miR-58 family miRNAs fine-tune EGL-1 activity and therefore critically contribute to the precise temporal control of programmed cell death during *C. elegans* development. We recently showed that, in the NSM lineage, EGL-1 activity is already present at a certain level in the NSM neuroblast and that this activity is necessary for cellular polarization of the NSM neuroblast and the segregation of apoptotic potential into the daughter that is programmed to die (for review, see Chakraborty et al. 2015; Lambie and Conradt 2016). Although we have not yet established that *egl-1* is necessary for cellular polarization in ABalappaap, we suggest that the copy number of *egl-1* mRNA needs to be kept not only below the threshold sufficient to trigger the mother's death but also above the threshold necessary to cause cellular polarization.

The observation that, apart from mothers, certain sisters inappropriately die in *mir-35* family mutants (“collateral” deaths) suggests that miR-35 and miR-58 family miRNAs may also be required for precise spatial control of programmed cell death during *C. elegans* development. In the ABalappaap and MSpaap lineages, the turnover of *egl-1* mRNA in the sisters programmed to live appears to occur mainly in a *mir-35* and *mir-58* family-independent manner; however, in some lineages, it may depend on miR-35 and miR-58 family miRNAs. Alternatively, collateral deaths may reflect the activity of lineage-specific regulators of *egl-1* transcription and their functional status, in this case, in the daughters programmed to survive.

Overall, we found 0%, 1.8%, and 8.0% of mothers of the first, second, and third waves of AB cell death to be affected by the loss of the *mir-35* family. Furthermore, based on our analysis of the ABalappaap lineage, the percentages for the second and third waves of cell death presumably increase twofold by the simultaneous loss of the *mir-58* family. Why are mothers of the first wave not affected and why are not more mothers of the second and third wave affected? Mothers of first-wave cell deaths may be resistant to the loss of the *mir-35* and *mir-58* family because other families of miRNAs may act to repress *egl-1* expression. Alternatively, an increase in *egl-1* expression at this developmental stage might not be sufficient to trigger deaths because the activities of other proapoptotic proteins (i.e., CED-4 and CED-3) might be limiting or because EGL-1 protein might be inactive due to post-translational control. The involvement of other families of miRNAs could also explain why not more mothers of second- and third-wave cell deaths die in *mir-35 mir-58* double-family mutants. Alternatively, in “resistant” mothers, as a result of lineage-specific control of *egl-1* transcriptional activation, the *egl-1* locus may simply not yet be transcribed. Finally, the cytoplasmic volume of mothers may play a critical role. The relatively large volume of mothers of first-wave cell deaths might confer a buffering capacity to modest changes in the transcriptome or proteome, and this buffering capacity might decrease with decreasing volume. In support of this notion, mothers of second-wave cell deaths, which are larger than mothers of third-wave cell deaths, appear to be more “resistant” than mothers of third-wave cell deaths.

### Conservation of miRNA-dependent control of programmed cell death

Through studies at single-cell resolution, we uncovered another aspect of the regulation of programmed cell death during *C. elegans* development: miRNA-dependent control of the activity of the BH3-only protein EGL-1. Interestingly, the *mir-35* family of miRNA genes is found only in other nematodes, although miRNAs with homologous functions might very well exist in higher animals, such as those reported to repress the mammalian BH3-only gene *Bim* (for review, see Sionov et al. 2015). However, the miR-58 family of miRNAs is homologous in both sequence and function to the *bantam* miRNA in *Drosophila*, which controls the expression of the proapoptotic gene *hid* (Brennecke et al. 2003). However, this conservation does not extend to higher animals. The conservation of miR-58 family miRNAs between nematodes and *Drosophila* hints at a similar developmental control of programmed cell death between the two model systems, which has been studied recently in depth (Li et al. 2014). It remains to be determined whether components of the core apoptosis pathway other than *egl-1* are also controlled by miRNA function during *C. elegans* development and whether single-cell studies will uncover additional aspects of the regulation of *egl-1* activity that may be conserved in *Drosophila* and mammals.

## Materials and methods

### General *C. elegans* maintenance and strains

*C. elegans* strains were cultured and maintained as described previously (Brenner 1974). The Bristol N2 strain was used as wild type, and the following transgenes and alleles were used in this study: LGI: *bcSi25* [P*_mai-2_gfp::h2b::mai-2 3′ UTR*] (this study), *bcSi26* [P*_mai-2_gfp::h2b::egl-1^wt^ 3′ UTR*] (this study), *bcSi27* [P*_mai-2_gfp::h2b::egl-1^mut mir-35^ 3′ UTR*] (this study), and *bcSi46* [P*_mai-2_gfp::h2b::egl-1^mut mir-58^ 3′ UTR*] (this study); LGII: *mir-35-41(nDf50)* (Miska et al. 2007), *mir-42-44(nDf49)* (Miska et al. 2007), *mIn1* [*mIs14 dpy-10(e128)*] (Edgley and Riddle 2001), and *bcSi47* [P*_mai-2_gfp::h2b::egl-1^mut mir-35 mir-58^ 3′ UTR*] (this study); LGIII: *mir-80(nDf53)* (Miska et al. 2007) and *unc-119(ed3)* (Maduro and Pilgrim 1995); LGIV: *mir-58.1(n4640)* (Miska et al. 2007) and *ced-3(n717)* (Shaham et al. 1999); LGV: *egl-1(n3330)* (B. Conradt); and LGX: *mir-81-82(nDf54)* (Miska et al. 2007). In addition, the following multicopy transgenes and extrachromosomal arrays were used: *nEx1187* [*mir-35 (genomic)+sur-5*::*gfp*] (Alvarez-Saavedra and Horvitz 2010) and *xdEx1091* [P_*unc-3*_*unc-3::gfp*+ P*_sur-5_rfp*] (Wang et al. 2015).

The single-copy integration alleles *bcSi25*, *bcSi26*, *bcSi27*, *bcSi46*, and *bcSi47* were generated using MosSCI (Frøkjær-Jensen et al. 2008) and the plasmids pBC1483, pBC1484, pBC1485, pBC1653, and pBC1654, respectively. The strain EG8078 [*oxTi185; unc-119(ed3)*] was used for targeted insertion on LGI, and EG6699 [*ttTi5605; unc-119(ed3)*] was used for targeted insertion on LGII (only pBC1654) (Frøkjær-Jensen et al. 2014).

### Plasmid construction

The plasmids pBC1483, pBC1484, and pBC1485 were constructed using a two-step overlap extension PCR and restriction enzyme cloning. First, P_*mai-2*_ was amplified as the 1763 base pairs (bp) immediately upstream of the *mai-2* start codon (Ichikawa et al. 2006), stopping at the transcription unit for B0546.4. The primers used for this were 5′-agatctGGAAAAAATCGATA-3′ (lowercase letters indicate BglII site) and 5′-GAAAAGTTCTTCTCCTTTACTCATTCTGAAAATTGAGTGAATTAG-3′. Second, this P_*mai-2*_ fragment was fused to the 1287-bp coding sequence of *gfp::h2b* as amplified from the plasmid pCM1.35 (a gift from G. Seydoux; Addgene plasmid no. 17248) using the primers 5′-CTAATTCACTCAATTTTCAGAATGAGTAAAGGAGAAGAACTTTT-3′ and 5′-GCTCGCGTTCTTGTACTGCAAATTACTTGCTGGAAGTGTACTTG-3′. Finally, the 3′ UTR from either *mai-2* (142 bp) or *egl-1*^*wt*^ (172 bp) was fused to P*_mai-2_gfp::h2b* to yield the final transgene. The *mai-2* 3′ UTR was amplified using the primers 5′-CAAGTACACTTCCAGCAAGTAATTTGCAGTACAAGAACGCGAGC-3′ and 5′-TcttaagTTCGCTAAAAACTA-3′ (lowercase letters indicate AflII site). The *egl-1*^*wt*^ 3′ UTR was amplified using the primers 5′-CAAGTACACTTCCAGCAAGTAAGTGATCAAAATCTCCAACTTTTC-3′ and 5′-TcttaagAAAAAAAACCATATTTATTATTAG-3′ (lowercase letters indicate AflII site). For the *egl-1*^*mut mir-35*^ variation, the wild-type sequence 5′-GATTTCTCATAATACCCGGT-3′ was mutated to 5′-CTAATCTCATACATCGCCGA-3′ using PCR site-directed mutagenesis and then validated by sequencing. The mutagenic primers used for this were 5′-TCTAATCTCATACATCGCCGAGTTTTTTCTTCATTTGTGATTATTTTTC-3′ and 5′-CTCGGCGATGTATGAGATTAGATGGTACAAATTGGAGAAAAG-3′. Each transgene was then ligated into the EcoRV site of pBluescript II KS^+^, subsequently excised using BglII/AflII, and ligated into the BglII/AflII site of the final destination MosSCI vector pCFJ350 (a gift from Erik Jorgensen; Addgene plasmid no. 34866) (Frøkjær-Jensen et al. 2012), which harbors the *cb-unc-119(+)* rescue fragment. The final plasmids were named pBC1483 (*mai-2* 3′ UTR transgene), pBC1484 (*egl-1*^*wt*^ 3′ UTR transgene), and pBC1485 (*egl-1*^*mut mir-35*^ 3′ UTR transgene).

The plasmids pBC1653 (*egl-1*^*mut mir-58*^ 3′ UTR transgene) and pBC1654 (*egl-1*^*mut mir-35 mir-58*^ 3′ UTR transgene) were constructed using PCR site-directed mutagenesis with the templates pBC1484 and pBC1485, respectively. The wild-type miR-58 family miRNA-binding site in the *egl-1* 3′ UTR was mutated from 5′-GAGAGATCGAAAA-3′ to 5′-CACTGTACCATAT-3′ using mutagenic primers 5′-CACTGTACCATATATAATCACAAATGAAGAAAAAACAC-3′ and 5′-ATATGGTACAGTGCGTCTCCAACTCCCCT-3′.

### 4D microscopy and lineage analysis

*C. elegans* L4 animals were grown to the adult stage overnight at 25°C. Two- or four-cell embryos were harvested from the young adults and mounted on 4.5% agarose pads for differential interface contrast (DIC) and fluorescence microscopy. 4D recordings were made throughout development as described previously (Schnabel et al. 1997, 2006) using a Zeiss Axio Imager.M2 and Time to Live software (Caenotec), capturing 25 DIC and/or fluroescence *z*-stacks every 35 sec at 25°C. Lineage analysis of the 4D recordings was performed using SIMIBioCell software (SIMI Reality Motion Systems, http://www.simi.com).

### smRNA FISH and image analysis

smRNA FISH was performed in *C. elegans* embryos, as described previously (Raj et al. 2008). Embryos were harvested by bleaching healthy adults and then permitted to develop in M9 buffer at 25°C until the desired stage was reached. Stellaris FISH probes (Biosearch Technologies) were designed against the mature mRNA of *egl-1* or *gfp*. The *egl-1* probe set contained 23 TAMRA-labeled oligonucleotides and was used at 250 nM. The *gfp* probe set to target *unc-3::gfp* contained 48 fluorescein-labeled oligonucleotides and was used at 500 nM. The *gfp* probe set to target *gfp::h2b* contained 30 TAMRA-labeled oligonucleotides and was used at 500 nM. Image stacks were obtained using a Leica LAS AF software and a Leica TCS SP5 II confocal microscope with a 63× oil immersion lens and a *z*-spacing of 500 nm to capture diffraction-limited mRNA spots over several *z*-slices.

Image analysis was performed using Fiji software (Schindelin et al. 2012). The number of nuclei in a given embryo was determined using Multiview Reconstruction software (Preibisch et al. 2010) to detect interest points in the DAPI channel, with manual corrections as required. The pipeline used to quantify the mRNA copy number in a cell of interest is outlined (Supplemental Fig. S5). Briefly, a three-dimensional region of interest (ROI) was defined for the cell of interest, and the background signal was subtracted. Next, the total smRNA FISH signal intensity was measured for this ROI from a *z*-projection summing the slices. Finally, the mRNA copy number for the cell of interest was calculated, dividing the total signal intensity of the ROI by the average intensity of a single diffraction-limited mRNA spot. For presentation, maximum intensity *z*-projection images were smoothed (Gaussian blur; radius, 1.5), and the DAPI signal of neighboring nuclei was removed. Quantification of *gfp::h2b* mRNA in transgenic embryos was performed on a maximum intensity *z*-projection of the smRNA FISH signal generated for the whole embryo. Next, a background subtraction (radius, 4) and Gaussian blur filter (radius, 1.5) were applied, and the resulting image was water-shedded. Finally, the number of particles was determined using the Analyze Particles tool (size, >15; circularity, 0–1). Cellular volumes were calculated by assuming sphericality and measuring the average diameter of the cell of interest from confocal image stacks.

## Supplementary Material

Supplemental Material
