# Supplementary material for: miRNAs cooperate in apoptosis regulation during C. elegans development
Source: Genes Dev. 2017 Jan 15;31(2):209–22. doi: 10.1101/gad.288555.116 (PMC5322734; doi:10.1101/gad.288555.116)
Supplement: Supplemental Material [file supp_31_2_209__index.html]

miRNAs cooperate in apoptosis regulation during C. elegans development — Supplemental Material 

# miRNAs cooperate in apoptosis regulation during *C. elegans* development

## Supplemental Material

- Supplemental\_Figures.pdf
