# Supplementary material for: miRNAs cooperate in apoptosis regulation during C. elegans development
Source: Genes Dev. 2017 Jan 15;31(2):209–22. doi: 10.1101/gad.288555.116 (PMC5322734; doi:10.1101/gad.288555.116)
Supplement: Supplemental Material [file supp_gad.288555.116_Supplemental_Figures.pdf]

SUPPLEMENTAL FIGURE LEGENDS

Figure S1. Programmed cell death in the AB lineage occurs in temporally distinct 'waves'. (*A*) A representative sub-lineage (ABala) of the *C. elegans* lineage tree (adapted from Sulston et al. 1983). Rounds of embryonic cell division (5th–11th) are indicated on the right. Cell deaths of the 1st wave are depicted in red, while those of the 2nd and 3rd waves are depicted in orange and yellow, respectively.

Figure S2. Large cell corpses in *mir-35* family mutants can be engulfed, persistent, or extruded. (*A*) Engulfment: a large cell corpse is engulfed by a neighboring cell over the course of 112 min. After this time, only granular remnants of the large cell corpse can be observed in the engulfing cell. (*B*) Persistence: a large cell corpse is still present in the embryo 120 min after forming. (*C*) Extrusion: A large cell corpse is extruded from the embryo after 90 min. Black scale bars: 2 µm; white scale bar: 10 µm. The following alleles were used: *mir-35-41(nDf50); mir-42 (nDf49).*

Figure S3. Blocking the apoptotic pathway fails to rescue embryonic lethality in *mir-35* family mutants. The terminal phenotype of a representative embryo is shown for each genotype, with an indication of either survival (hatched) or lethality (arrested). Scale bars: 10 µm. The following alleles were used: *mir-35-41(nDf50); mir-42 (nDf49); ced-3(n717)*; *egl-1(n3330)*.

Figure S4. The *egl-1* 3′UTR causes a reduction in mRNA copy number of the P*mai-2gfp::h2b* reporter. (*A*) Representative transgenic embryos are shown which express either the *mai-2* 3′UTR transgene or the *egl-1*^{wt} 3′UTR transgene in a +/+ genetic background. Embryos belong to one of three developmental stages (~50-cell, ~170-cell, and ~340-cell stage), and the smRNA FISH signal for *gfp::h2b* mRNA is shown together with DAPI (top images) or alone (bottom images). Scale bars: 10 µm. (*B*) Quantification of *gfp::h2b* mRNA in whole embryos at each stage of interest. n = 3–6 for each data point. Averages are plotted ± SEM.

Figure S5. Pipeline for the quantification of single-cell mRNA copy number using smRNA FISH. (*A*) The cell-of-interest is identified in whole-mount embryos by nuclear position, smRNA FISH staining, or a combination of the two. Once identified, a z-stack is captured through the cell with 500 nm spacing. A z-projection image is generated by summing all slices of the z-stack, and from this image the total smRNA FISH signal is measured. (*B*) The smRNA FISH signal from three identically sized, negative-staining regions are measured as background. These measurements are averaged and subtracted from the measurement in (*A*) to obtain the total smRNA FISH signal for the cell-of-interest. (*C*) Three distinct and independent "dots" are located and their total intensities measured as described in (A). An identically sized background measurement is subtracted from each, then all three measurements are considered to obtain the average intensity of a single mRNA. (*D*) Finally, the total smRNA FISH signal from (*B*) is divided by the average intensity of a single mRNA from (*C*), yielding the mRNA copy number for the cell-of-interest.

Figure S6. Cell-specific *egl-1* mRNA concentrations correlate with observed cell-death phenotypes in the ABalappaap and MSpaap lineages. (*A–B*) The mean *egl-1* mRNA concentration is plotted for mother and daughter cells of the (*A*) ABalappaap and (*B*) MSpaap lineages in the indicated genetic backgrounds (refer to legend for cell identity and genetic background). Cells are arranged by increasing *egl-1* mRNA concentration, for which the mean values are given above. Cells which always survive or die, regardless of the genetic background, are indicated. The following alleles were used: *mir-35-41(nDf50); mir-42 (nDf49); mir-80(nDf53); mir-58.1(n4640); mir-81-82(nDf54).*

Figure S7. Increased levels of *egl-1* mRNA in *mir* mutants do not accelerate the programmed death of MSpaapp (X). The time needed for MSpaapp to form a refractile corpse post-division was measured in each of the indicated backgrounds using 4D microscopy and lineage analysis. Averages are plotted ± SEM. The following alleles were used: *mir-35-41(nDf50); mir-42 (nDf49); mir-80(nDf53); mir-58.1(n4640); mir-81-82(nDf54).*

Figure S8. Temporal dynamics of *egl-1* mRNA copy number in the MSpaap lineage show a boost specifically in the MSpaapp (X) daughter. The copy number of *egl-1* mRNA is shown for the MSpaap cell and its two daughters (MSpaapa and Mspaapp [X]) over a developmental time-course. Shaded areas represent SEM, when applicable. The following alleles were used: *mir-35-41(nDf50); mir-42 (nDf49); mir-80(nDf53); mir-58.1(n4640); mir-81-82(nDf54).*

Figure S1

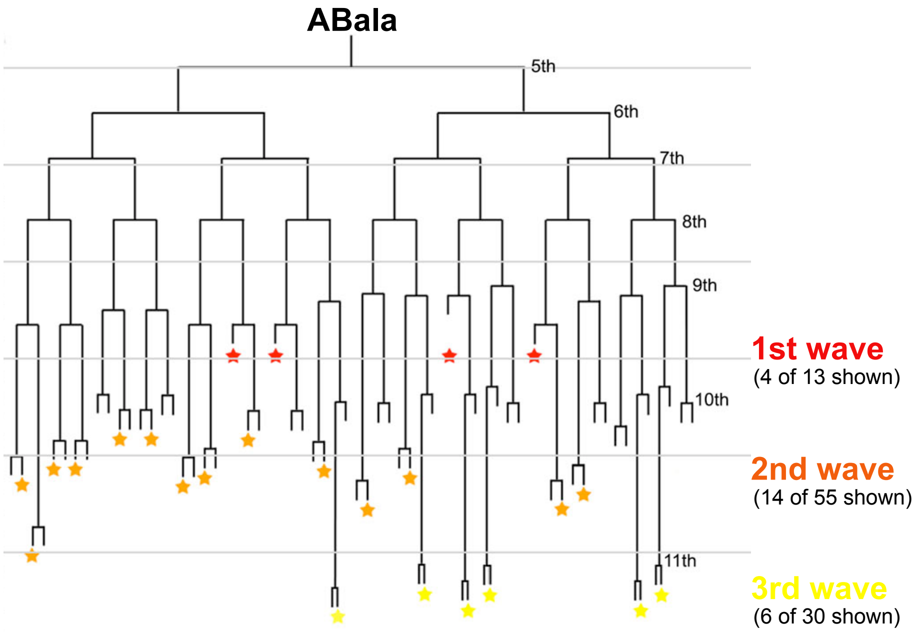

ABala
5th
6th
7th
8th
9th
10th
11th
1st wave
(4 of 13 shown)
2nd wave
(14 of 55 shown)
3rd wave
(6 of 30 shown)

Figure S2

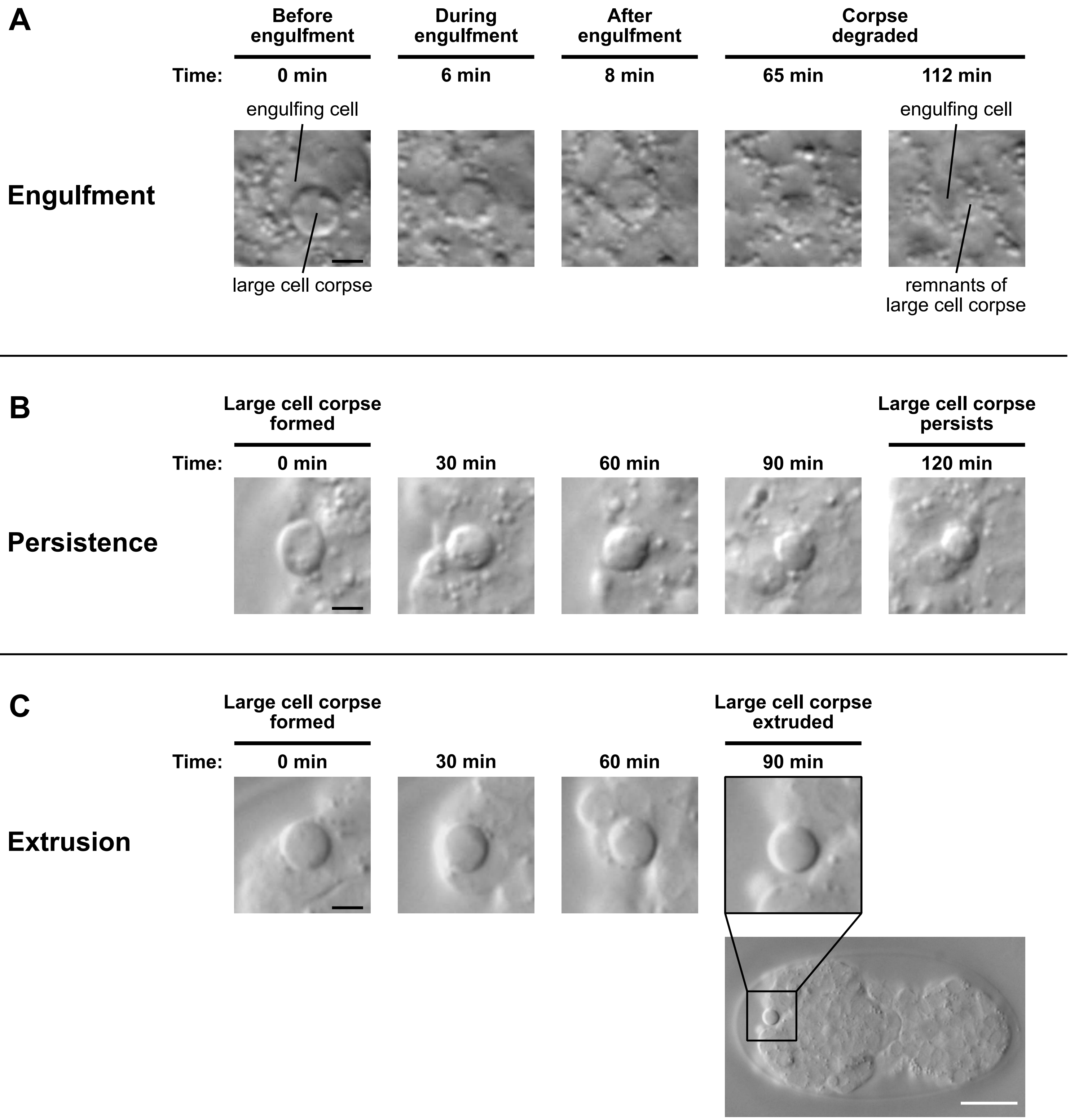
A
Before engulfment
During engulfment
After engulfment
Corpse degraded
Time:
0 min
6 min
8 min
65 min
112 min
engulfing cell
engulfing cell
Engulfment
large cell corpse
remnants of large cell corpse
B
Large cell corpse formed
Large cell corpse persists
Time:
0 min
30 min
60 min
90 min
120 min
Persistence
C
Large cell corpse formed
Large cell corpse extruded
Time:
0 min
30 min
60 min
90 min
Extrusion

Figure S3

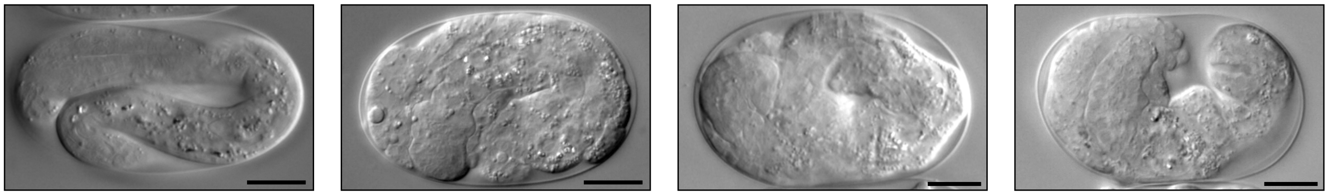
+/+
mir-35-42
mir-35-42; ced-3
mir-35-42; egl-1
hatched
arrested
arrested
arrested

Figure S4

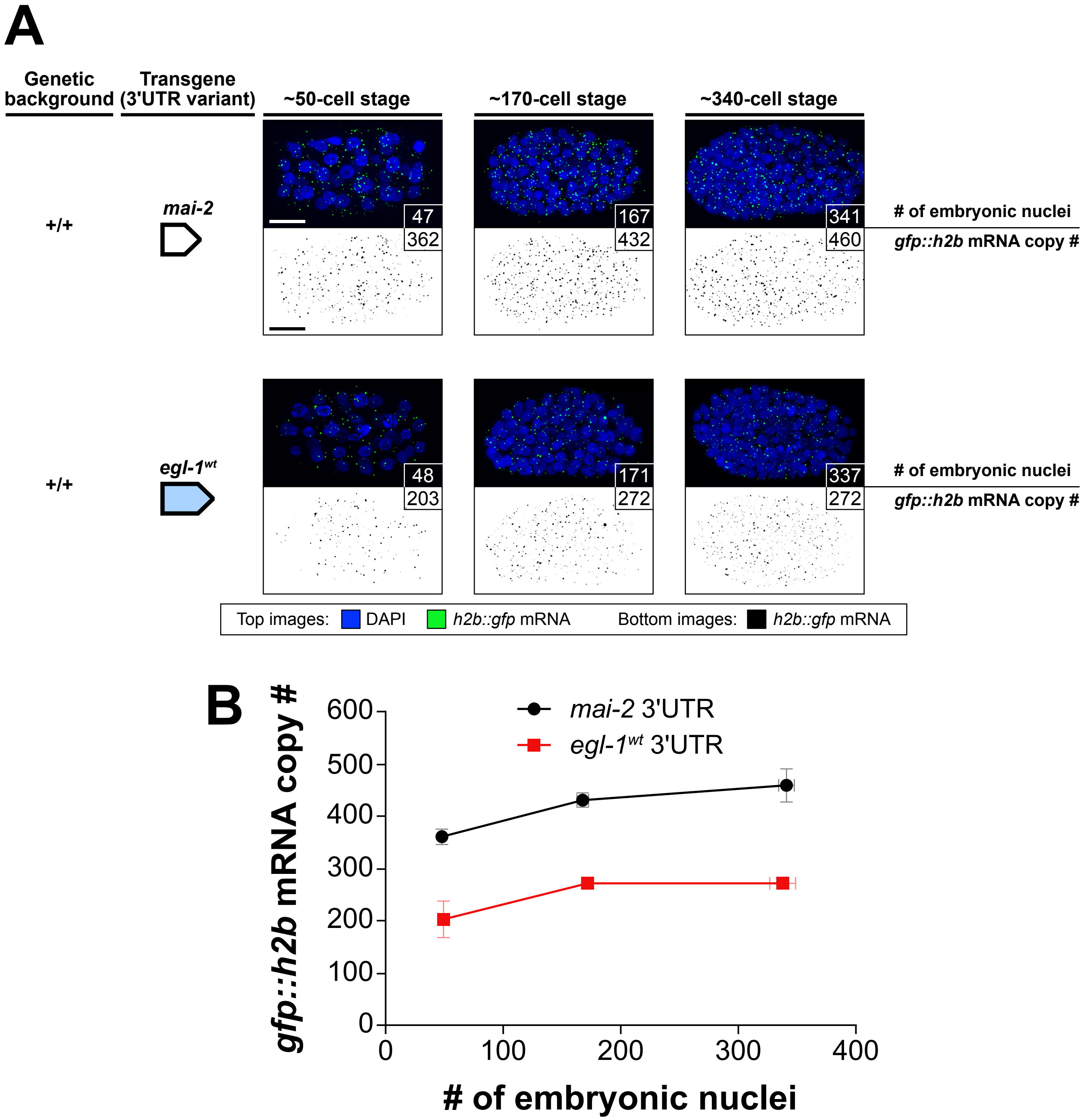

A
Genetic background
Transgene (3'UTR variant)
~50-cell stage
~170-cell stage
~340-cell stage
+/+
mai-2
47
362
167
432
341
460
# of embryonic nuclei
gfp::h2b mRNA copy #
+/+
egl-1wt
48
203
171
272
337
272
# of embryonic nuclei
gfp::h2b mRNA copy #
Top images: DAPI h2b::gfp mRNA Bottom images: h2b::gfp mRNA
B
mai-2 3'UTR
egl-1wt 3'UTR
gfp::h2b mRNA copy #
600
500
400
300
200
100
0
0
100
200
300
400
# of embryonic nuclei

Figure S5

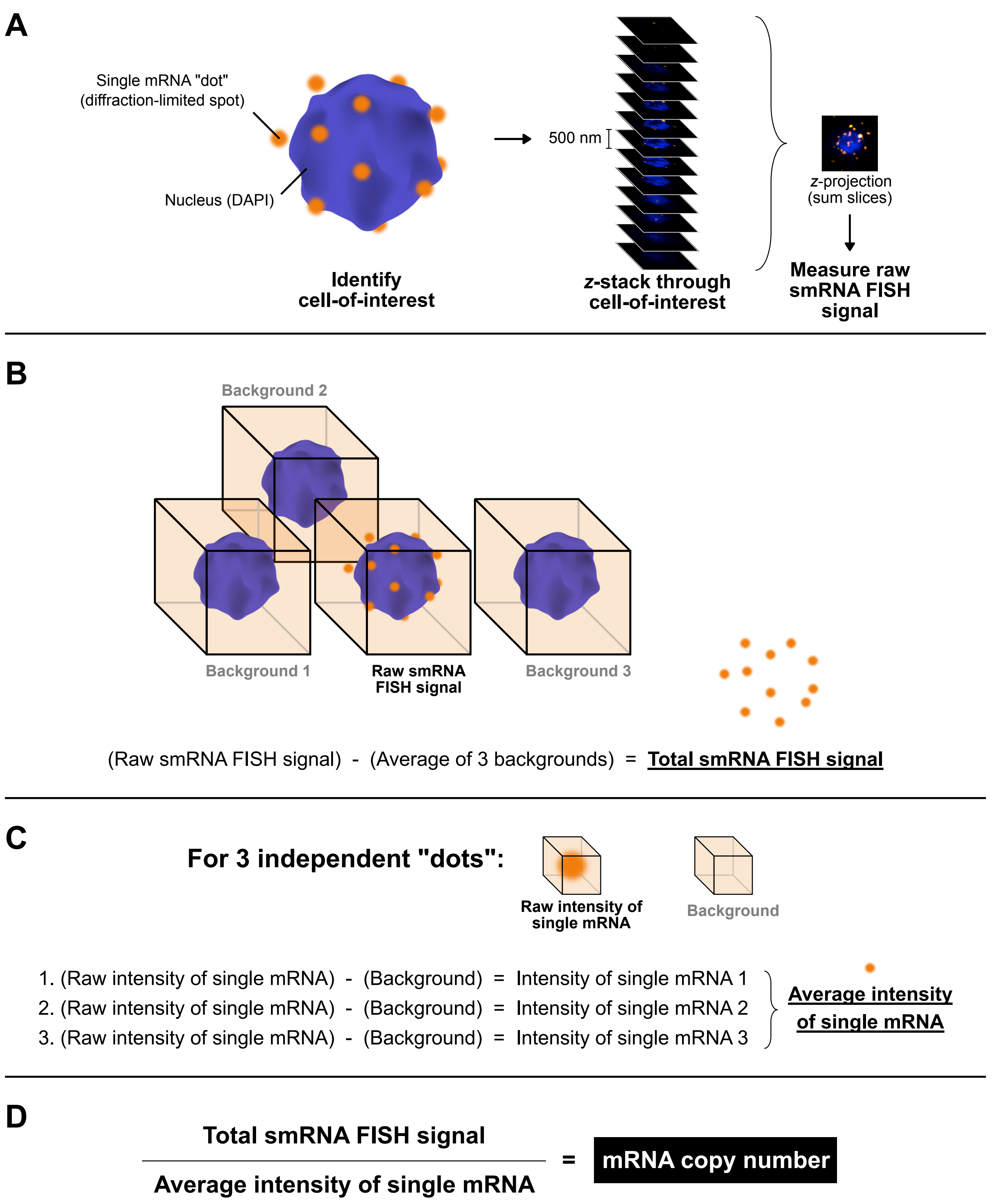
A
Single mRNA "dot"
(diffraction-limited spot)
Nucleus (DAPI)
Identify
cell-of-interest
500 nm
z-stack through
cell-of-interest
z-projection
(sum slices)
Measure raw
smRNA FISH
signal
B
Background 2
Background 1
Raw smRNA
FISH signal
Background 3
(Raw smRNA FISH signal) - (Average of 3 backgrounds) = Total smRNA FISH signal
C
For 3 independent "dots":
Raw intensity of
single mRNA
Background
1. (Raw intensity of single mRNA) - (Background) = Intensity of single mRNA 1
2. (Raw intensity of single mRNA) - (Background) = Intensity of single mRNA 2
3. (Raw intensity of single mRNA) - (Background) = Intensity of single mRNA 3
Average intensity
of single mRNA
D
Total smRNA FISH signal
Average intensity of single mRNA
=
mRNA copy number

Figure S6

A

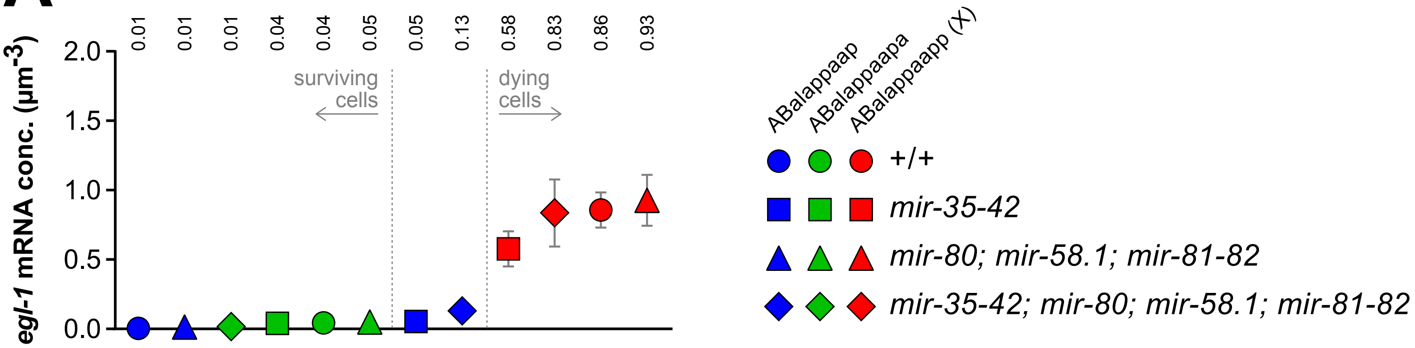
egl-1 mRNA conc. (µm-3)
0.01
0.01
0.01
0.04
0.04
0.05
0.05
0.13
0.58
0.83
0.86
0.93
2.0
1.5
1.0
0.5
0.0
surviving cells
dying cells
ABalappaap
ABalappaapa
ABalappaapp (X)
+/+
mir-35-42
mir-80; mir-58.1; mir-81-82
mir-35-42; mir-80; mir-58.1; mir-81-82

B

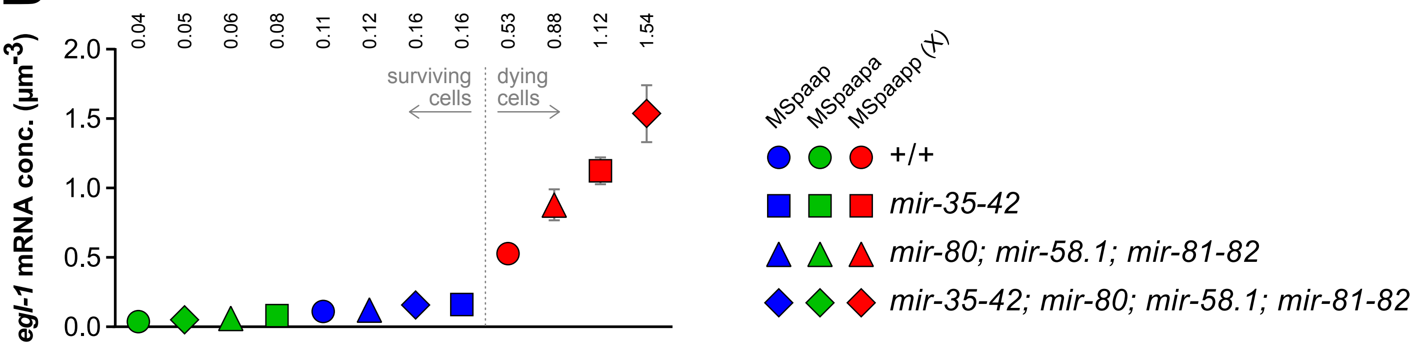
egl-1 mRNA conc. (µm-3)
0.04
0.05
0.06
0.08
0.11
0.12
0.16
0.16
0.53
0.88
1.12
1.54
2.0
1.5
1.0
0.5
0.0
surviving cells
dying cells
MSpaap
MSpaapa
MSpaapp (X)
+/+
mir-35-42
mir-80; mir-58.1; mir-81-82
mir-35-42; mir-80; mir-58.1; mir-81-82

Figure S7

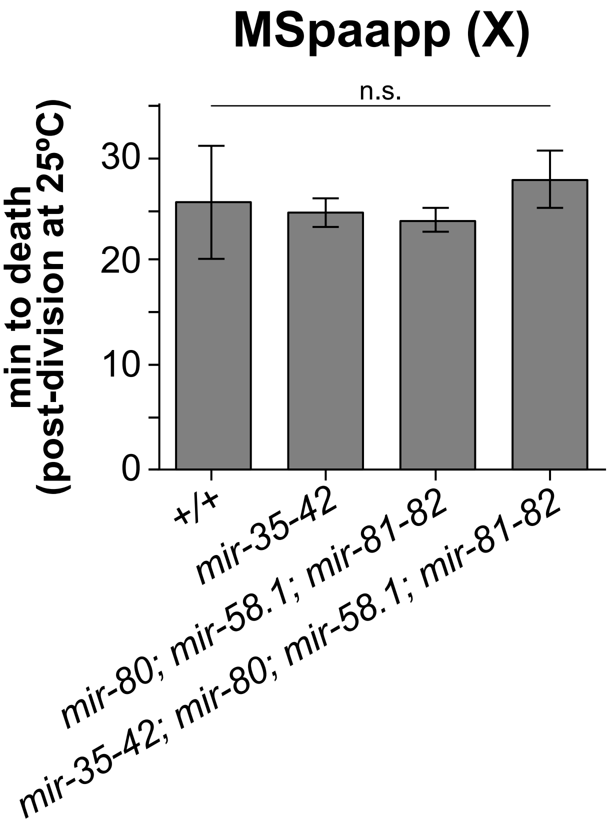
MSpaapp (X)
n.s.
min to death
(post-division at 25ºC)
30
20
10
0
+/+
mir-35-42
mir-80; mir-58.1; mir-81-82
mir-35-42; mir-80; mir-58.1; mir-81-82

Figure S8

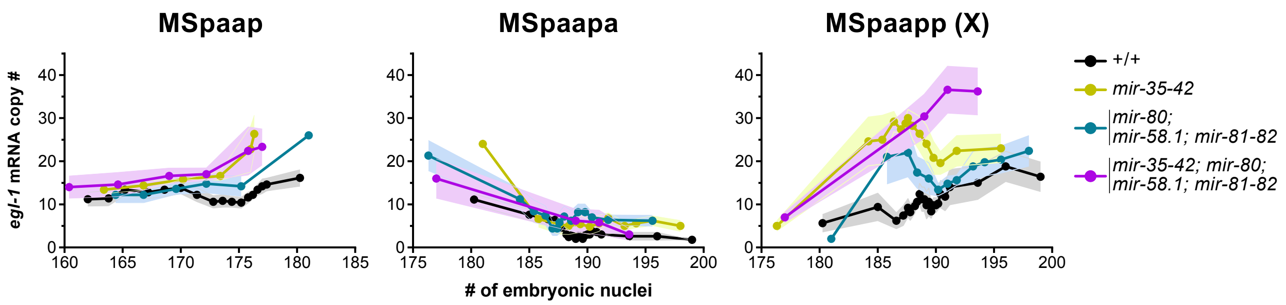

MSpaap
MSpaapa
MSpaapp (X)
egl-1 mRNA copy #
# of embryonic nuclei
0
10
20
30
40
160
165
170
175
180
185
175
180
185
190
195
200
+/+
mir-35-42
mir-80; mir-58.1; mir-81-82
mir-35-42; mir-80; mir-58.1; mir-81-82
